# Interplay among oncogenic pathways governs hepatobiliary lineage decisions in liver cancer

**DOI:** 10.1016/j.iliver.2026.100241

**Published:** 2026-05-06

**Authors:** Yonglong Pan, Xiaoping Chen, Ze-yang Ding

**Affiliations:** a Division of Hepato-Pancreato-Biliary Surgery, Hubei Key Laboratory of Hepato-Pancreatic-Biliary Diseases, Tongji Hospital, Tongji Medical College, Huazhong University of Science & Technology, Wuhan 430030, China; b Key Laboratory of Molecular Biophysics, Ministry of Education, College of Life Science and Technology, Huazhong University of Science and Technology, Wuhan 430074, China; c Key Laboratory of Organ Transplantation, Ministry of Education, National Health Commission, Chinese Academy of Medical Sciences, Wuhan 430030, China

**Keywords:** Liver cancer, RAS, Akt, Lineage conversion, Plasticity

Hepatocellular carcinoma (HCC), cholangiocarcinoma (CCA), and combined HCC–CCA have long been regarded as distinct pathological entities; however, accumulating evidence from lineage tracing, genomics, and transcriptomics increasingly supports the concept that liver cancers exist along a hepatobiliary continuum.^1^^,^^2^ The emergence of shared molecular subclasses between HCC and intrahepatic CCA (iCCA) highlights the remarkable plasticity of liver epithelial cells. It is essential to consider this evolving framework when interpreting studies on lineage conversion, including the recent work by Rösner et al., who reported that RAS/MEK/ERK (rat sarcoma viral oncogene homolog/ mitogen-activated protein kinase kinase/ extracellular signal-regulated kinase) activation drives biliary differentiation from hepatocytes.^3^

Rösner et al. recently reported in *Gut* that an elegant genetic model demonstrating that extracellular signal-regulated kinase (ERK) activation in hepatocytes induced biliary differentiation and promoted CCA-like malignancies.^3^ Their study provided interesting insights suggesting that oncogenic rat sarcoma viral oncogene homolog (RAS) signaling could redirect mature hepatocytes toward a biliary phenotype. However, several conceptual and mechanistic aspects of lineage determination in primary liver cancer deserve further consideration when interpreting these findings.

First, although the authors concluded that ERK signaling was a dominant determinant of biliary specification, the canonical biliary differentiation pathway Notch was not evaluated in their *Rb*^*lox/lox*^*;p53*^*lox/lox*^*;lsl-Kras*^*G12D*^ (*RPK);Alb*^*CrER*^ model. Notably however, hepatocyte-to-cholangiocyte conversion has long been recognized as a Notch-driven process.^4^ Canonical Notch1 signaling is both necessary and sufficient for biliary differentiation, and its induction produces tumors similar to those observed in the *RPK* model. In this context, the absence of canonical Notch readouts, including notch intracellular domain (NICD) accumulation and downstream targets such as hairy and enhancer of split-1 (Hes1) and hairy/enhancer-of-split related with YRPW motif protein 1 (Hey1), suggests that Notch signaling may contribute to the observed lineage switch. More broadly, these observations raise the issue of potential crosstalk between RAS–ERK and Notch pathways in regulating hepatobiliary fate decisions, although the nature and directionality of this interaction remain to be defined in the *RPK* model.

Second, the role of Akt in lineage decisions appears to be highly context-dependent and requires a more nuanced mechanistic interpretation. The authors propose that Akt maintains cellular dedifferentiation and supports iCCA development. Akt and signal transducer and activator of transcription 3 (STAT3) play critical roles in directing hepatic progenitor cells toward a hepatocyte-lineage fate.^5^ These divergent findings likely reflect differences in oncogenic and cellular contexts, including co-occurring pathway activation (such as mammalian target of Rapamycin complex 1 (mTORC1) signaling), metabolic state, the differentiation status of the target cell population, and microenvironmental influences. Nevertheless, the divergence highlights the need for a more nuanced interpretation of Akt function in hepatobiliary plasticity.

Third, the modelling outcomes reported by Rösner et al. differ from those observed in previous RAS-based liver cancer models. Hydrodynamic transfection-based studies using oncogenic RAS alone showed markedly different outcomes, with approximately 90% of the resulting liver tumors classified as HCC and only a minor fraction (∼10%) exhibiting mixed HCC/iCCA features.^6^ This discrepancy raises questions about the influence of the delivery method, genetic background, or microenvironmental perturbations. Reproducibility across models remains a crucial criterion when attributing deterministic lineage outcomes to specific oncogenic pathways. Finally, hepatocyte lineage stability constitutes a key biological constraint that influences the outcome of RAS-driven biliary conversion. Prior studies demonstrated that under simultaneous activation of hepatocytic differentiation programs, such as β-catenin or c-Myc, oncogenic RAS failed to induce biliary conversion and instead yielded HCC.^6^, ^7^, ^8^, ^9^ These observations highlight the fact that ERK activation alone is insufficient to override strong hepatocyte-fate cues. One possible interpretation is that RAS/ERK-driven biliary conversion may require the attenuation of hepatocyte lineage-stabilizing “molecular brakes”, potentially including master hepatocytic regulators such as hepatocyte nuclear factor 4 alpha (HNF4α), although this mechanism remains to be tested directly. Collectively, these findings indicate that lineage conversion in the liver is not dictated by isolated pathways, but emerges from the dynamic interplay between biliary-inducing (RAS, Notch, YAP, and SOX9) and hepatocyte-stabilizing (β-catenin and c-Myc) signals (Fig. 1).

**Fig. 1:**
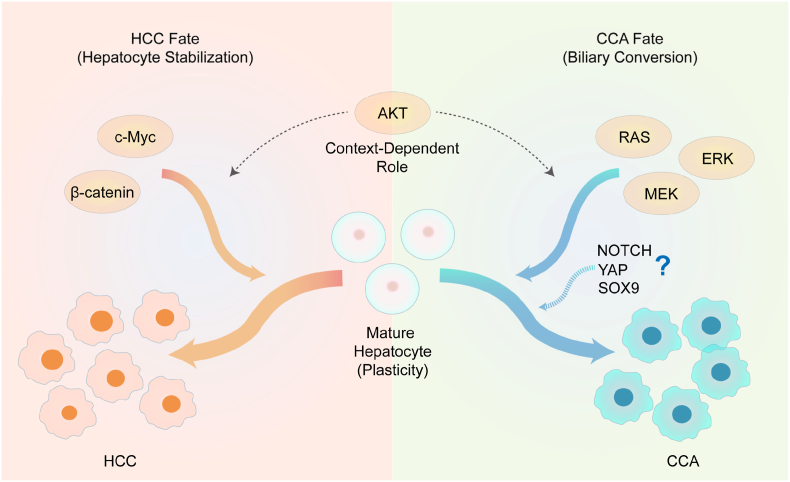
Interplay among oncogenic pathways governs hepatobiliary lineage decisions in liver cancer. Schematic representation illustrating plasticity of mature hepatocytes. Their fate is determined by the balance between biliary-inducing signals (RAS-ERK, and potentially Notch, YAP, SOX9) and hepatocyte-stabilizing programs (β-catenin, c-Myc). Strong activation of hepatocyte stabilizers can override RAS-driven conversion. The role of Akt remains context-dependent, contributing to lineage ambiguity. Abbreviations: HCC, hepatocellular carcinoma; CCA, cholangiocarcinoma; AKT, protein kinase B; c-Myc, MYC proto-oncogene, bHLH transcription factor; RAS, rat sarcoma viral oncogene homolog; ERK, extracellular signal-regulated kinase; MEK, mitogen-activated protein kinase kinase; YAP, yes-associated protein; SOX9, SRY-box transcription factor 9.

The study by Rösner et al. renews attention on the role of RAS/MEK/ERK signaling in biliary differentiation and liver cancer cell-fate decisions. Their findings contribute meaningfully to the ongoing effort to map the signaling determinants of hepatobiliary plasticity. However, more detailed mechanistic studies are needed to address the unresolved questions regarding Notch involvement, context-dependent Akt activity, model reproducibility, and the influence of competing lineage programs. Beyond tumor initiation, hepatobiliary lineage plasticity may have broader translational implications for liver cancer management. In regenerative clinical settings such as portal venous embolization,^10^ where compensatory liver remodeling occurs in both tumoral and non-tumoral tissues prior to resection, it will be important to determine how RAS/ERK and Notch signaling are engaged during liver regeneration and whether these processes influence tumor plasticity. More broadly, if RAS-driven biliary-like state transitions facilitate phenotypic adaptation, they may contribute to resistance to targeted and immune-based therapies, raising the possibility that future treatment strategies may need to combine pathway inhibition with approaches that constrain lineage plasticity or target biliary-like tumor states.

## CRediT authorship contribution statement

**Yonglong Pan:** Writing – original draft. **Xiaoping Chen:** Writing – review & editing. **Ze-yang Ding:** Writing – review & editing, Conceptualization.

## Informed consent

No written consent has been obtained from the patients as there is no patient identifiable data included.

## Data availability statement

The authors have nothing to report.

## Ethics statement

Not applicable.

## Declaration of generative AI and AI-assisted technologies in the writing process

During the preparation of this work the authors did not use generative AI or AI-assisted technologies.

## Funding

This research was funded by the 10.13039/501100001809National Natural Science Foundation of China (82303188 to Y.P., 82472970and 82273441to Z.D.).

## Declaration of competing interest

The authors declare that they have no known competing financial interests or personal relationships that could have appeared to influence the work reported in this paper.
